# Spectroscopic Characterization Using ^1^H and ^13^C Nuclear Magnetic Resonance and Computational Analysis of the Complex of Donepezil with 2,6-Methyl-β-Cyclodextrin and Hydroxy Propyl Methyl Cellulose

**DOI:** 10.3390/molecules30051169

**Published:** 2025-03-05

**Authors:** Nikoletta Zoupanou, Paraskevi Papakyriakopoulou, Nikitas Georgiou, Antigoni Cheilari, Uroš Javornik, Peter Podbevsek, Demeter Tzeli, Georgia Valsami, Thomas Mavromoustakos

**Affiliations:** 1 Department of Chemistry, National and Kapodistrian University of Athens, Zografou, 15771 Athens, Greece; nikolzoup@chem.uoa.gr (N.Z.); nikitage@chem.uoa.gr (N.G.); tzeli@chem.uoa.gr (D.T.); 2 Department of Pharmacy, National and Kapodistrian University of Athens, Zografou, 15771 Athens, Greece; ppapakyr@pharm.uoa.gr (P.P.); valsami@pharm.uoa.gr (G.V.); 3 Department of Pharmacognosy and Natural Products Chemistry, Faculty of Pharmacy, National and Kapodistrian University of Athens, Panepistimiopolis Zografou, 15771 Athens, Greece; cheilarianti@pharm.uoa.gr; 4 Slovenian NMR Centre, National Institute of Chemistry of Ljubljana, Hajdrihova 19, 1000 Ljubljana, Slovenia; uros.javornik@ki.si (U.J.); peter.podbevsek@ki.si (P.P.)

**Keywords:** donepezil, cyclodextrin, NMR, DFT

## Abstract

Donepezil (DH), a selective acetylcholinesterase inhibitor, is widely used to manage symptoms of mild to moderate Alzheimer’s disease by enhancing cholinergic neurotransmission and preventing acetylcholine breakdown. Despite the effectiveness of oral formulations, extensive hepatic metabolism and low systemic bioavailability have driven the search for alternative delivery systems. This study focuses on nasal delivery as a non-parenteral substitute, utilizing hydroxypropyl methylcellulose (HPMC) for its mucoadhesive properties and methyl-β-cyclodextrin (Me-β-CD) for its ability to enhance permeability and form inclusion complexes with drugs. Prior studies demonstrated the potential of HPMC-based nasal films for nose-to-brain delivery of donepezil and highlighted Me-β-CD’s role in improving drug solubility. Building on this, transparent gel formulations containing DH, HPMC, and 2,6 Me-β-CD were developed to investigate molecular interactions within two- and three-component systems. This study utilized a combination of nuclear magnetic resonance (NMR) spectroscopy and density functional theory (DFT) to provide detailed insights into the interactions between DH, 2,6-Me-β-CD, and HPMC. The findings provide critical insights into drug–excipient interactions, aiding the optimization of stability, solubility, and controlled release. This advances the rational design of nanotechnology-based drug delivery systems for enhanced therapeutic efficacy.

## 1. Introduction

Donepezil is among the most prescribed medications for the symptomatic treatment of mild to moderate Alzheimer’s disease (AD), which is characterized by cognitive decline and memory loss [1]. Its mechanism of action is based on the reversible inhibition of acetylcholinesterase (AChE), the enzyme responsible for breaking down acetylcholine in the synaptic cleft. By inhibiting this enzyme, donepezil increases acetylcholine concentration and prolongs its activity in the brain, thereby enhancing cholinergic neurotransmission [2]. Notably, donepezil exhibits high selectivity for acetylcholinesterase in the central nervous system (CNS), with minimal peripheral activity [3]. Its ability to cross the blood–brain barrier is facilitated by cation transporters but may be limited by p-glycoprotein (p-gp) efflux activity [4]. It has been shown to modulate nicotinic and muscarinic receptors, potentially contributing to its cognitive-enhancing effects [5]. Furthermore, donepezil may exhibit neuroprotective properties by reducing beta-amyloid plaque formation and oxidative stress, although these effects are secondary to its primary mechanism [6].

Chemically, donepezil is a benzylpiperidine derivative combined with a dimethoxy indanone ring. These structural features underline its activity as an acetylcholinesterase inhibitor [7]. Donepezil is commercially available as a racemic mixture for oral administration, containing equal amounts of its two enantiomers [8]. Following oral administration, donepezil exhibits good absorption properties, with peak plasma concentrations typically achieved within 3–4 h post-ingestion. It has a long half-life of about 70 h, allowing for once-daily dosing [9]. However, donepezil undergoes extensive first-pass metabolism in the liver, primarily mediated by cytochrome P450 enzymes CYP2D6 and CYP3A4, resulting in several metabolites, some of which retain AChE inhibitory activity [10]. Despite the high oral bioavailability of donepezil, the hepatic metabolism and slow absorption limit its effectiveness. These factors significantly reduce the fraction of the active drug that reaches systemic circulation unchanged [11]. Given these limitations, non-parenteral modes of administration are being explored to achieve more efficient delivery to the CNS with fewer systemic side effects. Namely, the development of donepezil nasal forms has been extensively studied, with various solid, liquid, and semi-liquid formulations reported in the literature [12,13].

Polymeric films represent a versatile dosage form applicable across various routes of administration, including buccal, ophthalmic, vaginal, and transdermal. In our previous study, the applicability of hydroxy propyl methyl cellulose (HPMC)-based nasal films for nose-to-brain delivery of donepezil was supported preclinically [14]. HPMC is widely utilized as a film-forming agent due to its hydrophilic nature, high swellability, and stability across a broad pH range [15]. The presence of both methoxy and hydroxypropyl groups, along with the flexibility of the cellulose backbone, facilitates effective interaction with mucin chains, resulting in high mucoadhesive properties [16,17].

The cyclic oligosaccharide methyl-β-cyclodextrin (Me-β-CD) is also extensively employed as a permeation enhancer in nasal delivery due to its ability to loosen the tight junctions of the nasal mucosa barrier [18]. The lipophilic cavity of CDs enables the formation of inclusion complexes with guest molecules, with multiple applications in drug delivery and food science [19]. Additionally, the external hydrophilic surface interacts with water molecules and can enhance the solubility of guest molecules [20]. Previous research has explored the inclusion of donepezil free base within the cavity of hydroxypropyl-β-cyclodextrin (HP-β-CD) using Fourier transform infrared spectroscopy (FT-IR) and molecular modeling techniques [21]. Despite previous studies on donepezil’s inclusion in HP-β-CD, its interaction with the more lipophilic Me-β-CD remains unexamined.

The combination of the two excipients, HPMC and Me-β-CD, has been shown to significantly increase the solubility of drugs such as carbamazepine and norfloxacin, probably due to the formation of a stable, more soluble, ternary complex [22,23]. Furthermore, the entanglement of HPMC molecules within the CD–drug network may act as a release modulator, potentially enabling the development of sustained-release formulations containing both polymers [24]. Nevertheless, the specific nature of this interaction, including the chemical groups involved, has not been fully understood.

In the context of our previous formulation study [25], transparent film-forming gels containing donepezil, HPMC, and 2,6 Me-β-CD were prepared. The current study builds upon these previous formulations by providing a detailed spectroscopic characterization of molecular interactions, which have not been previously reported. Specifically, it provides a detailed spectroscopic characterization of two-component and three-component systems, focusing on the potential molecular interactions between DH and the two excipients. To achieve this, we employed nuclear magnetic resonance (NMR) spectroscopy for detecting subtle changes in chemical environments. Also, density functional theory (DFT) was used in order to clarify the interactions between donepezil and cyclodextrin and also to compare with the experimental results. Specifically, to gain deeper insight into the interactions between cyclodextrin and donepezil, computational analysis was employed as a complementary approach. Molecular modeling will allow for a detailed characterization of binding modes, stability, and key intermolecular forces driving the complex formation. This theoretical study will enhance our understanding of the inclusion mechanism and provide a molecular-level perspective that supports the experimental findings. Understanding these interactions is considered critical, as they may influence the drug’s stability, solubility, and release profile within the delivery system.

## 2. Results and Discussion

**Figure 1:**
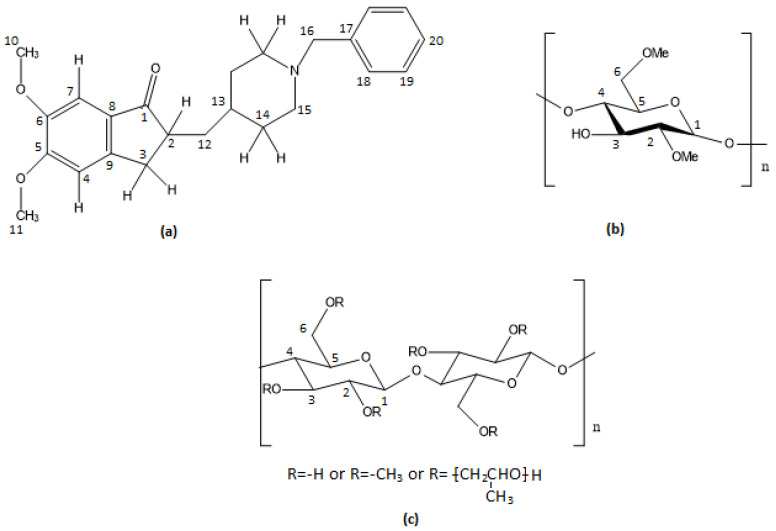
Structure of DH (**a**), 2,6-Me-β-CD (**b**), and HPMC (**c**).

The conformational analysis of molecules was accomplished via experimental and theoretical calculations. The first point of the analysis was the structural elucidation of donepezil (DH) (a), 2,6-Me-β-CD (b), and HPMC (c) in their normal and in complexed phase, using the acquired NMR spectra. Following this, DFT calculations were carried out in order to investigate the binding energy of atoms and their minimized conformations. The structure of examined molecules on which the spectroscopic and conformational study is based on are depicted in Figure 1 below.

### 2.1. NMR Analysis

#### 2.1.1. NMR Characterization of Donepezil (DH)

The structural characterization began by analyzing the spectra of donepezil, based on the received experimental DH results. The first molecular segment of DH identified was the aromatic protons, which resonated between 6.50 and 8.00 ppm in the ^1^H spectrum. This spectrum region included some overlapped peaks at 7.35–7.39 ppm and two peaks at 6.64 ppm and 6.57 ppm. According to the structure (Figure 1), the peaks resonating at 7.35–7.39 ppm correspond to H18-H19-H20, as they are the most deshielded protons of the molecule. The peaks at 6.64 ppm and 6.57 ppm were assigned to the H7 and H4 of DH prospectively. The assignment of these peaks was confirmed via their integration values, as well as the obtained homonuclear 2D-COSY and 2D-NOESY spectra. From the cross-peaks in the 2D HSQC-DEPT NMR spectrum, the corresponding carbon atoms were also identified.

For the remaining carbons, the assignment was carried out by studying the cross-peak correlations in the HSQC-DEPT spectrum. The coloring of the cross-peaks at this spectrum (phase positive/negative signals) differentiates the carbons based on the number of attached protons. For instance, peaks at 3.56 ppm and 3.64 ppm corresponded to the methyl protons of the 10 and 11 positions, due to the presence of oxygen atoms and the nearby aromatic group. The assignment of H10 and H11 was confirmed by the correlations shown in the NOESY spectrum with H7 and H4, correspondingly. The remaining signals at 2.25 ppm and 1.58 ppm were assigned to H2 and H13, respectively.

The H16 methylene group resonated at 4.14 ppm as it is situated in the deshielding environment of the phenyl ring and electronegative nitrogen. The peak identification continued with the rest of the protons at positions 3, 12, 14, and 15.

Correlations depicted in the HSQC-DEPT spectrum showed two double cross peak protons at 2.84/2.81 ppm and 2.33/2.29 ppm. In the HMBC spectrum, these protons were correlated with carbons C2, C7, and C1 (211.33 ppm), respectively. Based on the above data, protons with chemical shifts at 2.84/2.81 ppm and 2.33/2.29 ppm were identified as the diastereotopic protons of position 3, named H3 and H3′. Other signals observed were the protons at 1.08 ppm and 1.49 ppm, belonging to the same atom of carbon at 36.63 ppm according to the HSQC-DEPT spectrum. Moreover, their correlations with C2 and C13 at the HMBC spectrum and with H2 and H13 at the COSY spectrum led to their assignment, and they correspond to the diastereotopic H12 and H12′.

Diastereotopic H14 and H15 were the last examined protons of DH molecule. Peaks at 1.74–1.85 ppm and 1.26–1.32 ppm were observed to be correlated in the COSY spectrum with H13 and the protons resonating at 3.37 ppm and 2.88 ppm. The latter, not-assigned protons seemed to be correlated with H16 when studied at the HMBC spectrum. Therefore, signals at 3.37 ppm and 2.88 ppm corresponded to H15 and peaks at 1.74–1.85 ppm and 1.26–1.32 ppm corresponded to H14 and H14′, respectively. Their corresponding carbons were assigned by the HSQC-DEPT spectrum.

The final step was focused on the assignment of the quaternary carbons of the molecule. Clearly, C6, due to its chemical environment, was expected to correspond to the chemical shift of 155.38 ppm, while the next most deshielded carbons were those at positions 8 and 5. Based on the HMBC spectrum, it was found that C8 and C5 corresponded to 151.38 ppm and 148.15 ppm, respectively. According to the 2D HMBC experiment, the last quaternary carbons C17 and C9 were identified to resonate at 128.42 ppm and 127.19 ppm, respectively.

**Figure 2:**
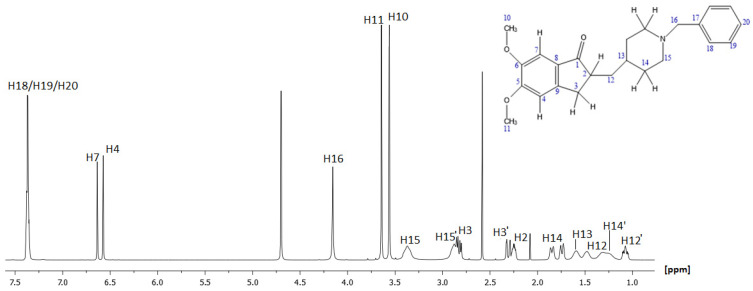
A 500 MHz ^1^H NMR spectrum of donepezil at 300 K using DMSO-*d*_6_ as solvent.

**Figure 3:**
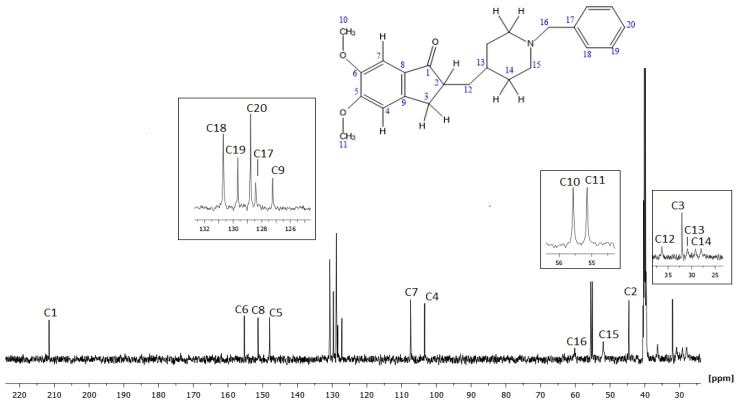
A 500 MHz ^13^C NMR spectrum of donepezil at 300 Κ using DMSO-*d*_6_ as solvent.

The full characterization of ^1^H and ^13^C spectra of DH is depicted in Figure 2 and Figure 3, while Table 1 records the assigned values of proton and carbon atoms expressed in ppm values. Assignment of the peaks are also reported in [26], with some discrepancies from our assignment; nevertheless, our detailed and comprehensive analysis through 1D and 2D spectra did not permit any ambiguity in our results.

#### 2.1.2. NMR Characterization of 2,6-Methyl-β-Cyclodextrin (2,6-Me-β-CD)

**Figure 4:**
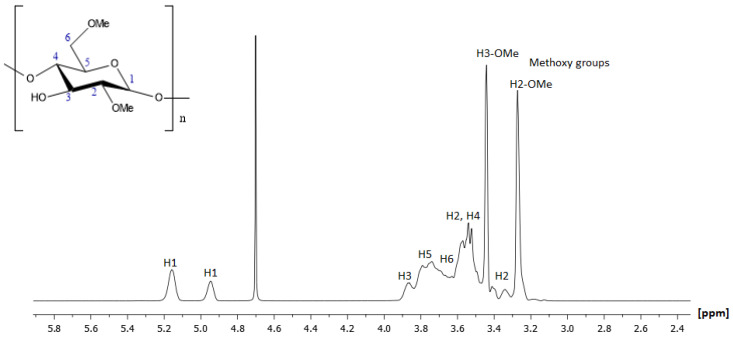
A 500 MHz ^1^H NMR spectrum of *2,6-Me-β-CD* at 300 K using D_2_O as solvent.

**Figure 5:**
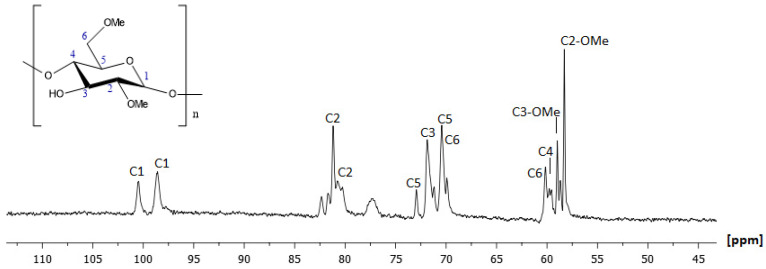
A 500 MHz ^13^C NMR spectrum of *2,6-Me-β-CD* at 300 K using D_2_O as solvent.

The study proceeded with the analysis of the supermolecule of 2,6-Me-β-CD. The assignment started with the identification of the anomeric proton of position H1, as is the most deshielded proton of the molecule. On the ^1^H NMR spectrum, it resonated at 5.16 ppm and 4.95 ppm, indicating the two possible conformers. The assignment of the molecule was completed by analyzing the correlations of the vicinal protons in the COSY spectrum. The ring protons (H2, H3, H4, H5, and H6) were expected to have signals in the region from 3.20 to 4.20 ppm. H3 and H5 showed chemical shifts slightly upfield in comparison to H2 and H4. Their corresponding carbon atoms were identified by the HSQC-DEPT spectrum. The structure elucidation of 2,6-Me-β-CD was confirmed via all the remaining obtained homo- and heteronuclear spectra and a comparison with similar studies [27]. Figure 4 and Figure 5 depict the ^1^H and ^13^C NMR spectra of 2,6-Me-β-CD with all the assigned peaks. The chemical shifts were expressed in ppm for protons and carbons, and they are referred to in detail in Table 2.

#### 2.1.3. NMR Characterization of Hydroxypropyl Methyl Cellulose (HPMC) 

NMR spectroscopy provided detailed insights into the cellulose ring and the functional groups of HPMC, including hydroxyl (–OH), methoxy (–OCH_3_), and hydroxypropyl (–CH_2_CHOHCH_3_) groups. Proton NMR revealed broad signals and overlapped peaks due to the polymeric nature of HPMC, as expected from the nature of the compounds, with distinct peaks associated with the methoxy and hydroxypropyl groups.

**Figure 6:**
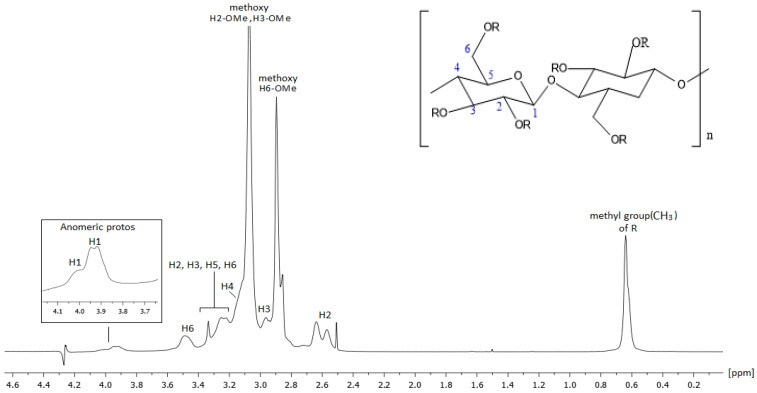
A 500 MHz ^1^H NMR spectrum of HPMC at 300 K using DMSO-*d*_6_ as solvent.

**Figure 7:**
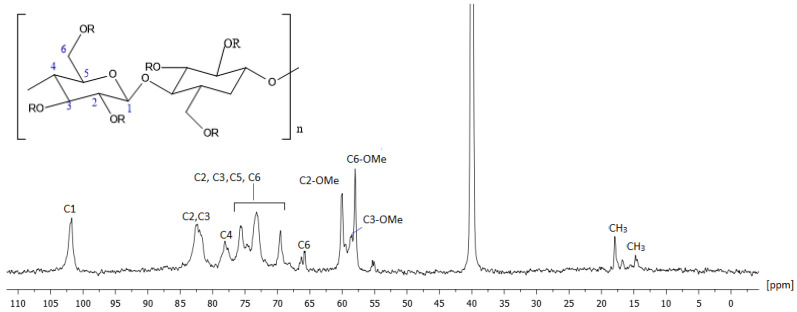
A 500 MHz ^13^C NMR spectrum of HPMC at 300 K using DMSO as solvent.

^1^H and ^13^C NMR peaks were characterized based on the references reported in the literature and our 500 MHz homonuclear and heteronuclear spectra. Our results were in agreement with the literature [28,29]. The ^1^H and ^13^C spectra are presented in Figure 6 and Figure 7, respectively, and all the ppm values of proton and carbon atoms of the molecule are recorded in detail in Table 3.

#### 2.1.4. NMR Analysis of the Complex

A necessary part of the study involved the confirmation of the complexation of the three compounds: DH, 2,6-Me-β-CD, and HPMC. After the structure elucidation of each molecule separately, the procedure continued by studying their observed chemical shifts in single and complexed conditions. The results of a sample of P1 were compared with the results of the mixtures of P4, P5, and P7. Only some of the peaks of proton atoms of molecules **a**, **b**, and **c** were able to be observed in the complexed phase, due to the overlapping of peaks. The chemical shifts are recorded in Table 4, Table 5 and Table 6.

The below conclusions can be derived from Table 4. When DH was mixed in 2,6 Me-β-CD generally, an upfield shift of the signals was observed when compared with DH alone. This clearly indicates that DH was engulfed in the interior hydrophobic cavity of CD. The interaction of DH with the cavity of CD, resulting in a water-stable inclusion complex, has been observed through FT-IR spectroscopy [21] in the case of complexation with HP-β-CD. Notably, molecular modeling revealed hydrogen bonding between the carbonyl as well as the tertiary nitrogen groups of DH and the hydroxyl groups of HP-β-CD, accompanied by moderate interaction energy. In contrast, the interaction between DH and 2,6 Me-β-CD predominantly involves hydrophobic interactions, as the secondary hydroxyl groups in HP-β-CD were replaced with methyl groups in Me-β-CD, reducing hydrogen bonding potential. Similar hydrophobic effects were observed when DH was mixed with HMPC. Interestingly, the interactive effect of CD relative to HPMC was higher in most of the cases, suggesting its stronger interaction with DH, as well as a preference of DH for CD over HPMC when all three molecules coexist in the formulation. The entanglement of DH with the HPMC chains is further supported by the FT-IR analysis conducted by Monou et al., which revealed the disappearance of characteristic peaks in the presence of HPMC suggesting that the drug molecules are effectively incorporated into the polymer matrix. In the cases of H4 and H11, CD is shown to exert a higher effect, indicating that this segment of DH is in the spatial vicinity of an even more hydrophobic segment of HPMC than the 2,6 Me-β-CD. The most upfield signal effect was observed with H15, indicating that this proton interacts with the most hydrophobic environment, either of the CD or HPMC.

The presence of DH in the trimolecular mixture including HPMC and CD caused further upfield shifts. This was indicative that DH experienced a hydrophobic environment. Thus, DH interacted mainly with the hydrophobic environment of both CD and HPMC.

It appears that two equilibria were taking place:


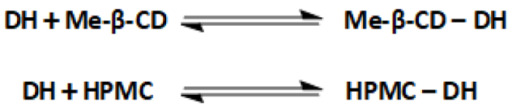


The trimolecular complexing can be also confirmed by noticing that there was an increase of Δν_1/2_ of the peaks constituting the trimolecular complex. Such an increase of Δν_1/2_ is expected as the motion of DH was restrained.

**Figure 8:**
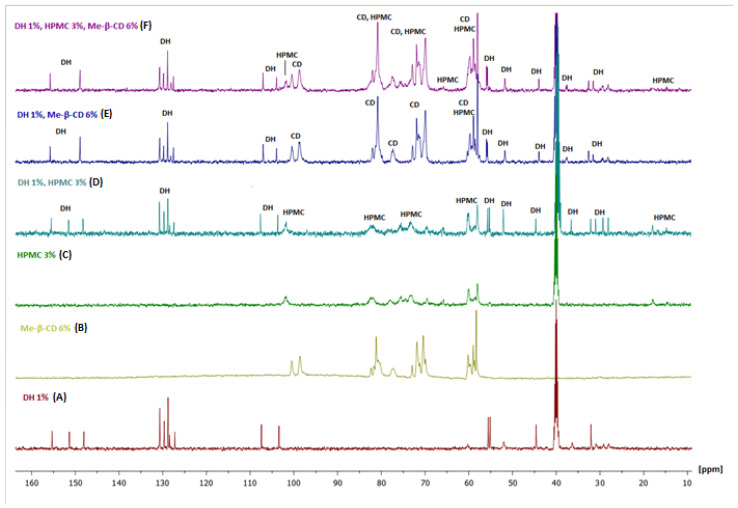
Superimposed spectra of DH 1% (**A**), Me-β-CD 6% (**B**), HPMC 3% (**C**), DH 1%-HPMC 3% (**D**), DH 1%-Me-β-CD 6% (**E**), and DH 1%-HPMC 3%-Me-β-CD 6% (**F**) at 300 K using a 500 MHz NMR spectrometer.

Below in Figure 8 are the ^13^C NMR spectra of the six samples studied.

##### Mixture of DH and HPMC

The complexation with HPMC was weak, as can be observed in the DH-HPMC biomolecular mixture (Figure 8D). The peaks of DH (see Figure 8 spectrum A and D) kept the same linewidth in all aromatic regions. Moreover, the three peaks around 30 ppm (C13, C14) showed increased intensity and shorter Δv_1/2_, indicating higher flexibility than DH alone.

##### Mixture of Me-β-CD and DH

In contrast with the previous mixture, the main characteristic of the biomolecular mixture was that the peak at 151.35 ppm disappeared, clearly indicating the complexing of DH carbonyl with the Me-β-CD (see Figure 8A,E). It was also evident that no increase of flexibility was observed with DH peaks. The three peaks around 30 ppm showed the same intensity and linewidth as the DH alone.

##### Mixture of Me-β-CD, HPMC and DH

A few important observations were outlined in the trimolecular complex (Figure 8F): (a) Me-β-CD predominated the interactions. Thus, the peak at 151.35 ppm disappeared again. (b) The region at 30 ppm resembled DH (Figure 8A,F). Only small differences were observed.

### 2.2. DFT Calculations

**Figure 9:**
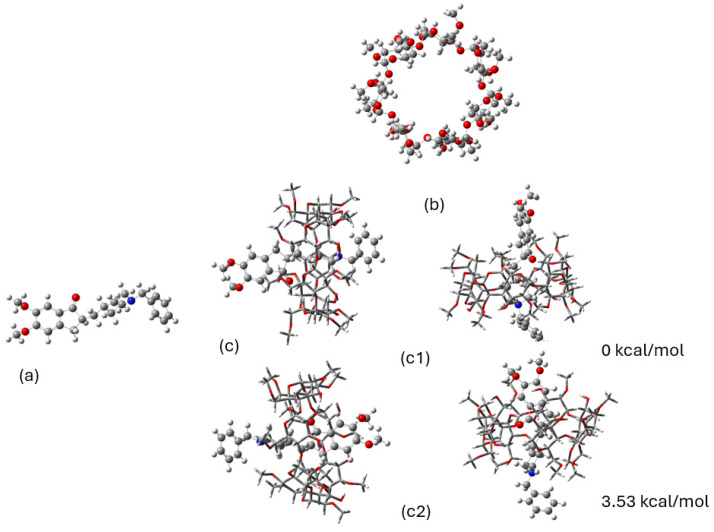
Calculated minimum energy structures: (**a**) donepezil, (**b**) 2,6Μe-β-CD, and (**c**) complex of donepezil with 2,6Μe-β-CD (**c1**) and complex of reversed donepezil with 2,6Μe-β-CD (**c2**) from two different points of view. All calculations were performed in DMSO solvent using B3LYP/6-31G(d,p). The energy differences between the conformations in complexes are given.

The DFT calculations (B3LYP/6-31G(d,p)) in the DMSO environment yielded the minimum energy conformations for donepezil, 2,6Me-β-CD, and the corresponding donepezil–CD complex, as depicted in Figure 9. This methodology was appropriate for the calculation of the encapsulation of molecules CD, since our previous experimental and theoretical studies on quercetin encapsulation in 2-hydroxyl-propyl-β-cyclodextrin (2HP-β-CD) and 2,6-methylated-β-cyclodextrin (2,6Me-β-CD) [30], as well in CD dimeric complexes, i.e., in (2HP-β-CD)_2_ and (2,6Me-β-CD)_2_, have shown that the calculated ^1^H NMR spectra and UV–VIS absorption spectra were in very good agreement with our experimental data.

**Figure 10:**
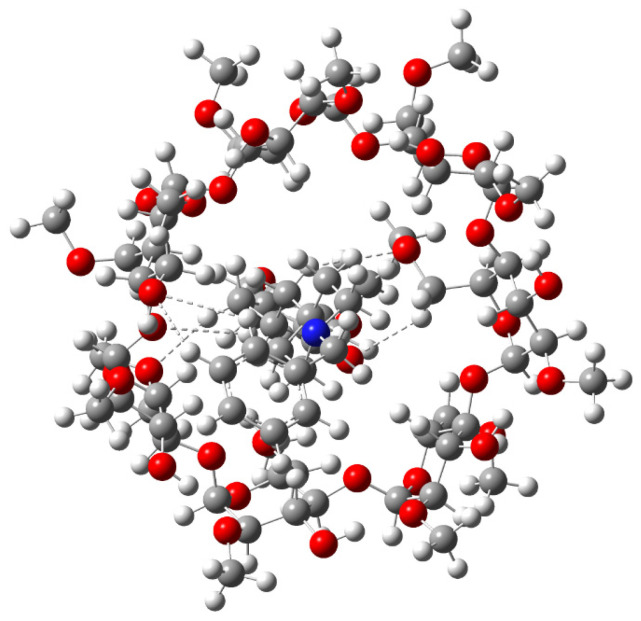
Conformations of the donepezil complexed with 2,6Me-β-CD. Hydrogen bonds are shown in dashed lines.

For the present calculations, one unit of 2,6Me-β-CD was used. Donepezil was encapsulated into the CD cavity in two different directions. Two minimum structures of the complexes were calculated: (i) the piperidine interacting with the methoxy and -OH rim of the CD (**c1** complex shown in Figure 1) and (ii) the dimethoxy group interacting with the methoxy and -OH rim of 2,6Me-β-CD (**c2** complex shown in Figure 1). The **c1** complex was pocessed the lowest energy, and **c2** was 3.53 kcal/mol higher in energy than **c1**. Figure 10 illustrates the hydrogen bonds between donepezil and 2,6Me-β-CD. In this case, six hydrogen bonds were formed within the 2.9 Å range. It is crucial to emphasize that binding energies of H...O interactions exceeding 2.9 Å are nearly negligible [31]. The complexation energies and the deformation energies of the 2,6Me-β-CD and donepezil molecules are detailed in Table 6.

The calculated binding energies for the complex are presented in Table 6. The deformation energy of the encapsulated donepezil was 0.5 kcal/mol, showing a very small deformation energy, while the deformation energy of 2,6Me-β-CD was 7.29 kcal/mol, with 12.38 kcal/mol to increase the binding interaction of CD with donepezil. The binding energies (BEs) of the deformed structures of the complex component, denoted as BEr, were −9.77 kcal/mol for **c1** and −8.17 kcal/mol for **c2**. It should be noted that the **c2** complex was not stable with respect to the minimum free structures of the complex components; however, the **c1** complex remained stable with respect to them, showing that the complex can be isolated, making it suitable for NMR studies and observing the structural changes of the complex in comparison to the isolated molecules.

**Figure 11:**
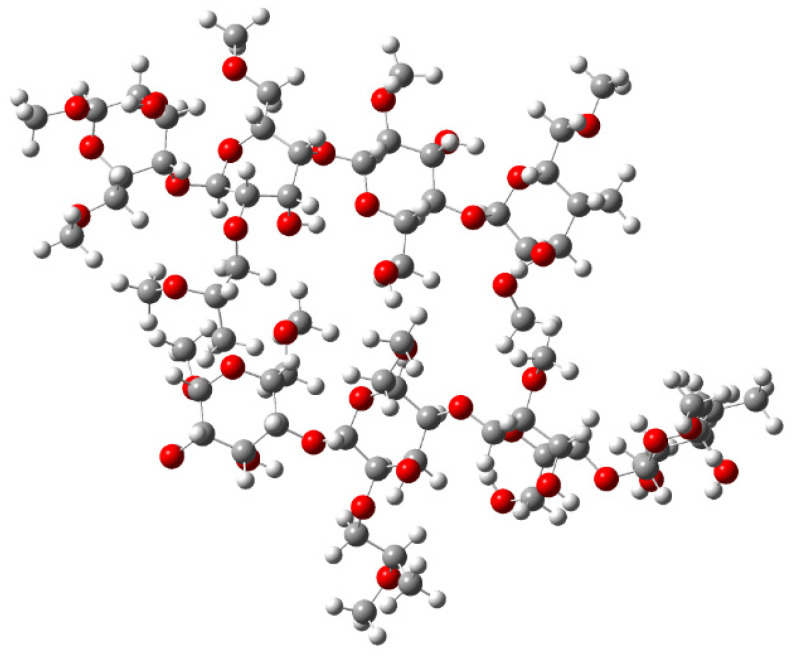
Minimum conformation structure of HPMC using B3LYP/6-31G(d,p).

The lowest conformation of HPMC was calculated using the B3LYP/6-31G(d,p) method (see Figure 11).

### 2.3. Study of NOEs of Molecules in Complexed Phase

The NMR experimental procedure included the acquisition of the NOESY spectrum of molecules a, b, and c in complex conditions, detecting NOE correlations among them. DH and HPMC molecules presented a relative interaction, with specific correlations of some of their proton atoms appearing in space. The through-space interactions within the two molecules were confirmed by the signal of aromatic protons of 18/19/20 position and H4 of DH with the methoxy groups of the HPMC molecule. H7 of DH seemed to interact with the ring protons of HPMC too.

## 3. Materials and Methods

### 3.1. Chemicals

Donepezil hydrochloride (DH, MW: 379.50 g/mol, analytical grade) was obtained from Cipla Ltd. (Mumbai, India), and methyl-β-cyclodextrin (Me-β-CD, MW: 1310 g/mol, pharmaceutical grade) was procured by Fluka Chemika (Mexico City, Mexico and St. Louis, MO, USA). Hydroxy propyl methyl cellulose (HPMC, Methocel E50 premium LV, MW: 90,000 g/mol, pharmaceutical grade) and poly (ethylene gly-col) 400 (PEG 400, pharmaceutical grade) were purchased from Colorcon (Shanghai, China) and Sigma Chemical Company (St. Louis, MO, USA), respectively. Triple-deionized water from Fischer Scientific (Waltham, MA, USA) (HPLC grade) was used for all preparations. Dimethyl sulfoxide-*d*_6_ (deuteration degree) >99.8% and deuterated water (D_2_O) for NMR spectroscopy was purchased from Sigma-Aldrich (St. Louis, MO, USA).

### 3.2. Preparation of Samples

To investigate potential interactions of DH with the two excipients (HPMC and Me-β-CD), different formulations were prepared and subjected to both one- and two-dimensional spectroscopy experiments. The composition of the formulations in terms of excipients and drug concentrations was investigated in our previous study [25], and the highest concentration levels of each excipient was used in this study to assess potential interactions among them. For DH and 2,6 Me-β-CD solutions (P1, P3), 100 mg of DH and 600 mg of 2,6 Me-β-CD were separately dissolved in 10 mL of water, achieving concentrations of 1% *w/v* for DH and 6% *w/v* for Me-β-CD. For the DH-Me-β-CD solution (P5), both amounts were combined in the same volume. In gel preparations containing HPMC (P2, P4, P6, P7), methocel E50 served as the gel-forming polymer at a concentration of 3% *w*/*w*. The manufacturing process of HPMC-based gels was carried out by following the standard protocol of dispersion of HPMC in hot water (>80 °C). After dispersion, the mixture was cooled to below 10 °C and maintained for 15 min to ensure complete hydration [25]. DH (1% *w*/*w*) and 2,6 Me-β-CD (6% *w*/*w*) were added either separately or together to explore potential two-component or three-component interactions. Specifically, weighed amounts of HPMC E50 were dispersed in hot distilled water along with Me-β-CD, under continuous magnetic stirring (600 rpm). The homogeneous mixture was subsequently cooled to 15 °C to facilitate polymer hydration. Finally, weighed quantities of DH were added to achieve a concentration of 1% (*w*/*w*) in the resulting gel (Table 7). 

### 3.3. NMR Experiments

The samples were analyzed by means of nuclear magnetic resonance (NMR) spectroscopy, and NMR experiments were carried out at the NMR Core Facilitiy of the National and Kapodistrian University of Athens on a Bruker Avance NEO 500 MHz system (magnet ASCEND 11.76 Tesla) operating at 500.11 MHz for ^1^H and 125.75 for ^13^C and controlled by TopSpin 4.1.4. Experiments were acquired at 300 K, and temperature was controlled by a Bruker BCU II Cooler unit. A total of 15 mg of donepezil (DH), 2,6-methyl-beta-cyclodextrin (2,6-Me-β-CD), and hydroxypropyl methyl cellulose (HPMC) and their complex was diluted in 750 μL of DMSO-d6 and placed in 5 mm NMR tubes (Sigma Aldrich). Due to the limited solubility of 2,6-Me-β-CD in DMSO-*d*_6_, NMR experiments were repeated in D_2_O solvent. Samples were measured with a double resonance broadband inverse (BBI) detection probe or a broadband observe (BBO H&F) Prodigy cryoprobe (Bruker, Germany). The 1D (^1^H, ^13^C) and 2D homo- and heteronuclear experiments (COSY, NOESY, HSQC-DEPT, and HMBC) were acquired with Bruker’s standard pulse programs. In the case of the HMPC sample, ^1^H, COSY, NOESY, and HSQC-DEPT spectra were acquired with pressaturation of the residual water signal with optimization of the ^1^H 90° pulse length. For 2D NOESY experiments, various mixing times (d8) were applied from 0.15 to 0.8 s. The spectroscopic NMR data were analyzed and processed using standard Bruker NMR software (TopSpin 3.5) and MestreNova 14.2 software.

### 3.4. Density Functional Theory (DFT) Calculations

Density functional theory (DFT) calculations were employed to assess the interactions between donepezil and 2,6Me-β-CD. Initially, donepezil was optimized using the B3LYP [32,33]/6-31G(d,p) [34] method to identify its lowest energy structure. Additionally, conformational analysis [35] was conducted for 2,6Me-β-CD to determine its lowest energy structures using the B3LYP/6-31G(d,p) method. Specifically, for the CD, 7 units were used for the conformational analysis. This methodology has been used in similar complexes [36]. Subsequently, the complexes formed by CD and donepezil were optimized to identify the lowest minima. Also, the HPMC polymer was optimized using B3LYP/6-31G(d,p). All calculations were carried out in a DMSO solvent, employing the polarizable continuum model (PCM) [37]. Gaussian 16 was utilized for all calculations and for the visualization of the results [38].

## 4. Summary and Conclusions

For the first time, NMR spectroscopy and density functional theory (DFT) were combined to study the DH-2,6-Me-β-CD complex. DFT calculations provided compelling evidence that DH binds to cyclodextrin with an affinity of −9.77 kcal/mol. Quantum calculations used in this study revealed the establishment of hydrogen bonds between donepezil and 2,6 Me-β-CD. Specifically, six hydrogen bonds were observed, ranging from 2 to 2.9 Å. The NMR data are in accordance with DFT findings, clearly indicating the intercalation of DH into the hydrophobic core of 2,6 Me-β-CD. A similar complexation of DH was also observed with HPMC. When DH was combined with both excipients in the formulation, a distinct preference of DH with 2,6-Me-β-CD over HPMC was observed. These findings suggest that 2,6-Me-β-CD is the preferred excipient for DH due to its stronger binding affinity and favorable supramolecular interactions. However, if 2,6 Me-β-CD is to be included in a more lipophilic environment, a combination of HPMC and 2,6 Me-β-CD appears to provide the optimal formulation environment.

This study offers valuable insights into DH’s molecular interactions within novel formulations, guiding the development of nanotechnology-based drug delivery systems. Understanding these interactions enables the rational design of optimized drug–excipient complexes for enhanced therapeutic performance. Such studies are instrumental in driving the design of new drug formulations by leveraging an in-depth understanding of drug–supramolecule interactions at the molecular level.

## Figures and Tables

**Table 1:** Chemical shifts of atoms of protons and carbons of donepezil expressed as ppm values.

Position	Protons (ppm)	Carbons (ppm)	Position	Protons (ppm)	Carbons (ppm)
H1	-	211.33	H11	3.64	55.24
H2	2.25	44.82	H12	1.49	36.63
H3	2.84/2.81	32.42	H12′	1.08	36.63
H3′	2.33/2.29	32.42	H13	1.58	31.29
H4	6.57	103.22	H14	1.85/1.74	29.58
H5	-	148.15	H14′	1.22/1.31	28.24
H6	-	155.38	H15	3.37	52.21
H7	6.64	107.48	H15′	2.88	52.21
H8	-	151.38	H16	4.16	60.63
H9	-	127.24	H17	-	128.42
H10	3.56	55.79	H18/H19/H20	7.35–7.39	131.05/129.15

**Table 2:** Chemical shifts of atoms of protons and carbons of 2,6-Me-β-CD expressed as ppm values.

Position	Protons (ppm)	Carbons (ppm)
H1	5.16	98.67
H1′	4.95	100.54
H2	3.52	80.59
H2	3.34	81.62
H3	3.87	71.83
H4	3.54	59.02
H5	3.79	72.97
H6a	3.73	60.13
H6b	3.62	70.49
H6-O-Me	3.44	58.98
H2-O-Me	3.27	58.29

**Table 3:** Chemical shifts of atoms of protons and carbons of HPMC expressed as ppm values.

Position	Protons (ppm)	Carbons (ppm)
H1	3.94	101.75
H1	4.01	101.94
H2	2.57	82.44
H2	2.64	82.01
H3	2.96	82.48
H2, H3, H5, H6	3.24, 3.12, 3.03, 3.22, 3.14, 3.11	69.50, 73.22, 74.50, 75,49, 75.49, 78.04
H4	3.12	78.08
H4	3.14	77.63
H6	3.44	66.27
H6	3.49	65.82
-H6-OCH_3_	2.86	55.03
-H6-OCH_3_	2.90	58.00
H2-OCH_3/_H3-OCH_3_	3.06	58.53
H2-OCH_3/_H3-OCH_3_	3.07	60.05
H2-OCH_3/_H3-OCH_3_	3.28	59.33
H2-OCH_3/_H3-OCH_3_	3.45	59.43
-OH	4.91	-
-OH	5.04	-
R(-CH_3_)	0.62	14.60
R(-CH_3_)	0.62	16.48
R(-CH_3_)	0.64	17.90

**Table 4:** Chemical shifts of protons of DH in normal and complexed phases as ppm values.

Position of P1 Protons	Chemical Shifts of P1	Chemical Shifts of P5	Chemical Shifts of P4	Chemical Shifts of P7	Δδ of DH (P1–P4)	Δδ of DH (P1–P5)	Δδ of DH (P1–P7)
H18, H19, H20	7.35–7.39	6.98–7.01	6.97–6.99	6.97–7.01	0.38–0.36	0.38–0.36	0.38–0.36
H7	6.64	6.53	6.45	6.53	0.19	0.11	0.11
H4	6.57	6.40	6.44	6.41	0.13	0.17	0.16
H16	4.16	3.79	3.76	3.79	0.4	0.37	0.37
H10	3.56	3.37	3.27	3.46	0.29	0.19	0.10
H11	3.64	3.32	3.34	3.38	0.3	0.32	0.26
H12	1.08	0.80	0.80	0.80	0.28	0.28	0.28
	1.49	1.25	1.19	1.28	0.3	0.24	0.21
H14	1.74–1.85	1.45–1.51	1.35–1.51	1.44–1.52	0.29–0.33	0.29–0.34	0.3–0.08
	1.26–1.32	1.03	0.92	1.02	0.24	0.23	-
H13	1.58	1.30	1.24	1.31	0.27	0.28	0.3
H2	2.25	2.15	2.08	2.11	0.17	0.10	0.14
H15	3.37	-	2.51	2.37	0.86	-	1
	2.88	2.43	-	-	-	0.45	-
H3	2.84/2.81	2.80–2.77	2.62	2.37–2.49	0.04	0.04	0.47–0.32
	2.33/2.29	2.15–2.18	2.12	2.14–2.18	0.21	0.18	0.19–1.1

**Table 5:** Carbon atoms of DH alone and their chemical shifts in mixtures of P4, P5, and P7.

Position of P1 Protons	Chemical Shifts of P1	Chemical Shifts of P5	Chemical Shifts of P4	Chemical Shifts of P7
C1	211.33	207.80	211.66	207.85
C6	155.38	155.75	155.48	155.74
C8	151.38	-	151.52	-
C5	148.15	148.92	148.24	148.91
C18	130.69	130.70	130.77	130.72
C19	129.67	129.80	129.73	129.83
C20	128.79	128.87	128.83	128.88
C17	128.42	128.13	128.39	128.10
C9	127.24	127.55	127.43	127.55
C7	107.48	107.02	107.65	107.05
C4	105.41	103.97	103.68	103.96
C10	55.79	55.94	55.69	55.94
C11	55.24	55.77	55.29	55.76
C2	44.82	43.90	44.68	43.91
C3	32.42	31.56	31.01	31.55
C15	52.21	51.75	52.10	51.76
C12	36.63	37.48	36.57	37.55
C14	29.58	29.29	29.32	29.44
C14′	28.24	28.22	28.11	28.09
C16	60.63	Overlappted peak	60.17	Overlappted peak

**Table 6:** Binding energies with respect to the minimum structure conformers of the components of the complexes, BE, and with respect to the complexed structures of the components of the complex, BEr, as well as deformation energies of donepezil and cyclodextrin, DefD and DefCD, at B3LYP/6-31G (d, p). All energies are in kcal/mol.

Donepezil@2,6Me-β-CD	c1	c2
BE_r_	−9.77	−8.17
BE	−2.00	+4.59
Def_D_	0.48	0.38
Def_CD_	7.29	12.38

**Table 7:** Composition of the samples.

Preparation	DH	HPMC	Me-β-CD
	% *w*/*w*	% *w*/*w*	% *w*/*w*
P_1_	1		−
P_2_	−	3	−
P_3_	-		6
P_4_	1	3	−
P_5_	1	−	6
P_6_	−	3	6
P_7_	1	3	6

## Data Availability

The data presented in this study are available in the present article.
